# Human seven-β-strand (METTL) methyltransferases - conquering the universe of protein lysine methylation

**DOI:** 10.1016/j.jbc.2023.104661

**Published:** 2023-03-29

**Authors:** Pål Ø. Falnes, Jędrzej M. Małecki, Maria C. Herrera, Mads Bengtsen, Erna Davydova

**Affiliations:** Department of Biosciences, Faculty of Mathematics and Natural Sciences, University of Oslo, Oslo, Norway

**Keywords:** chaperone, DOT1L, methyltransferase, METTL, mitochondria, seven-β-strand, protein lysine methylation, S-adenosylmethionine, translation elongation factor

## Abstract

Lysine methylation is an abundant posttranslational modification, which has been most intensively studied in the context of histone proteins, where it represents an important epigenetic mark. Lysine methylation of histone proteins is primarily catalyzed by SET-domain methyltransferases (MTases). However, it has recently become evident that also another MTase family, the so-called seven-β-strand (7BS) MTases, often denoted METTLs (methyltransferase-like), contains several lysine (K)-specific MTases (KMTs). These enzymes catalyze the attachment of up to three methyl groups to lysine residues in specific substrate proteins, using *S*-adenosylmethionine (AdoMet) as methyl donor. About a decade ago, only a single human 7BS KMT was known, namely the histone-specific DOT1L, but 15 additional 7BS KMTs have now been discovered and characterized. These KMTs typically target a single nonhistone substrate that, in most cases, belongs to one of the following three protein groups: components of the cellular protein synthesis machinery, mitochondrial proteins, and molecular chaperones. This article provides an extensive overview and discussion of the human 7BS KMTs and their biochemical and biological roles.

The function of many proteins is regulated or optimized by posttranslational methylation. A substantial number of enzymes and other proteins are involved in introducing, removing, and recognizing protein methylation, which plays a key role in the regulation and optimization of numerous cellular and physiological processes. Protein methylation occurs primarily on lysine and arginine residues, but recent research suggests that also histidine methylation is pervasive (1, 2, 3, 4, 5).

Protein lysine methylation is mediated by methyltransferase (MTase) enzymes that catalyze the transfer of a methyl group from the methyl donor AdoMet to a lysine residue in the target protein. The sequential action of these lysine (K)-specific MTases (KMTs) can introduce up to three methyl groups on each lysine, yielding the three distinct methylation states monomethyllysine, dimethyllysine, and trimethyllysine (Kme1, Kme2, Kme3) (Fig. 1*A*). Methylation does not affect the positive charge of lysine but increases its bulkiness and hydrophobicity (6). In mammals, protein lysine methylation was reported already in 1964, when it was discovered that histone proteins contain methyllysine (7). Subsequent studies during the 80s and 90s revealed that several other proteins are subject to lysine methylation. These included many abundant and important proteins, such as chaperones of the 70 kDa heat shock protein (Hsp70, aka HSPA) family (8), calmodulin (CaM) (9), eukaryotic translation elongation factor 1α (eEF1A) (10), muscle myosins (11), as well as the mitochondrial proteins ATP synthase (ATPS) (12) and citrate synthase (CS) (13). However, the KMTs responsible for these methylations remained elusive for several decades.

**Figure 1:**
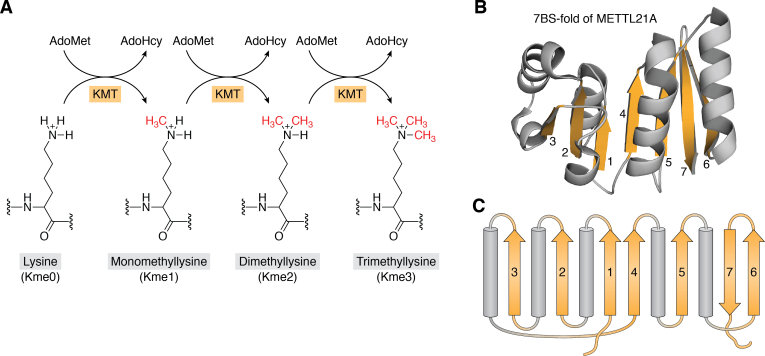
**The KMT reactions and the 7BS-fold.***A*, reactions catalyzed by lysine-specific MTases (KMTs). *S*-adenosylmethionine (AdoMet) acts as methyl donor and is converted to its unmethylated counterpart, *S*-adenosylhomocysteine (AdoHcy) during the reaction. *B*, cartoon representation of the 7BS-fold of the KMT METTL21A (218 amino acids). Only the 7BS region (amino acids 68–211) is shown. The β-strands are shown in *orange* and numbered; α-helices are shown in *gray*. The structure (PDB: 4LEC) was visualized using PyMOL Molecular Graphics System, Version 2.4.1. (Schrodinger, LLC) (https://pymol.org/). *C*, schematic representation of the classical 7BS topology. Color code and numbering as in *B*.

The field of lysine methylation gained traction when the first KMTs responsible for histone methylation were discovered, thus enabling functional studies on specific histone lysine methylations (14). Further advances included the discoveries of a number of so-called reader domains capable of recognizing specific histone lysine methylations and of demethylases reversing the methylations (15, 16). This was paralleled by the development of methods for studying histone methylation status genome-wide (17). Thus, histone lysine methylation has now been established as a highly important and dynamic set of epigenetic modifications playing essential roles in regulating gene expression and chromatin state. The first discovered histone KMTs belong to a distinct MTase family, the so-called SET-domain proteins, named after the founding members Suppressor of variegation 3-9 (Su(var)3-9), Enhancer of zeste (E(z)), and Trithorax (Trx) (14, 18). The SET-domain family, with about 50 human members, constitutes the second largest MTase family in humans and includes all the KMTs involved in lysine methylation of flexible histone tails (18, 19).

Interestingly, it was found that a non-SET-domain KMT, DOT1L (Dot1-like), was responsible for methylation of K79 in the globular part of histone H3 (20, 21, 22). DOT1L belongs to the so-called seven-β-strand (7BS) MTase family (also denoted "Class I" MTases), which has ∼130 human members, and represents the largest MTase family (19, 23). The 7BS MTases share a three-dimensional structure containing a characteristic seven-stranded β-sheet (or variations thereof), as well as certain conserved motifs involved in AdoMet binding (23, 24, 25). These motifs have been described in detail in previous articles, and certain mutations within them invariably abrogate MTase activity. The β-sheet of the 7BS MTases has a distinct order and topology, with most of the β-strands separated by α-helices, and all but the last of them (β7) running in the same direction (Fig. 1, *B* and *C*) (23, 25). Collectively, the 7BS MTases methylate a wide range of substrates, including DNA, RNA, proteins, and small molecules (25). The sequence similarity between different 7BS MTases is often low and limited only to the short, degenerate motifs involved in AdoMet binding. However, several of the human 7BS MTases form distinct subgroups of interrelated proteins that target similar substrates and show higher sequence similarity, particularly in regions involved in substrate recognition (19, 23).

Since the discovery of DOT1L, numerous additional human 7BS KMTs have been uncovered and all of these target nonhistone proteins (26, 27, 28, 29, 30, 31, 32, 33, 34, 35, 36, 37, 38, 39, 40, 41, 42, 43, 44). Interestingly, the majority of these 7BS KMTs were found to catalyze one of the nonhistone lysine methylations reported in the early studies mentioned above, and where the responsible KMT had remained elusive for decades (28, 29, 30, 31, 32, 34, 35, 37, 38, 39, 42, 43). We published in 2016 a review on 7BS KMTs in all three kingdoms of life, encompassing also several human enzymes (23). Since then, however, considerable progress has been made, with the number of human 7BS KMTs almost doubled, meriting a review focused on these enzymes. In the first part of this article, we provide an overview of the human 7BS KMTs and briefly describe the individual enzymes, whereas in the second part, various aspects of this enzyme family will be discussed in a more general manner.

## Overview and scope

**Figure 2:**
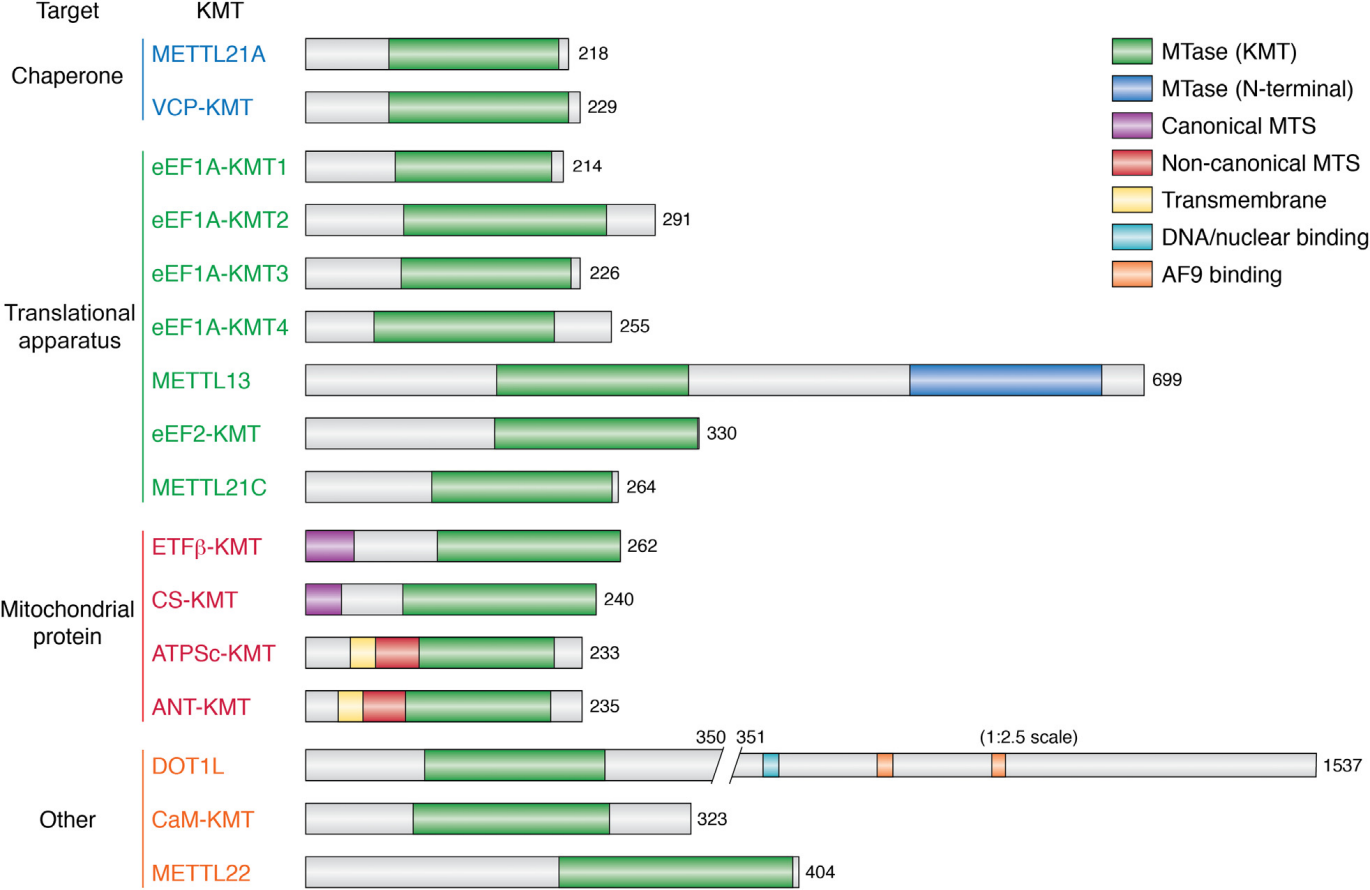
**Overview anddomain architecture of human 7BS KMTs.** The enzymes have been grouped according to the type of substrate they target. The core 7BS domain is shown in *green* and other domains are colored as indicated. MTS, mitochondrial targeting sequence. Whereas the first MTase domain of METTL13 catalyzes lysine methylation of eEF1A, the second MTase domain (*dark blue*) is an eEF1A N-terminal MTase. 7BS, seven-β-strand; eEF1A, eukaryotic translation elongation factor 1α; KMT, lysine-specific MTase; METTL, methyltransferase-like; MTase, methyltransferase.

**Figure 3:**
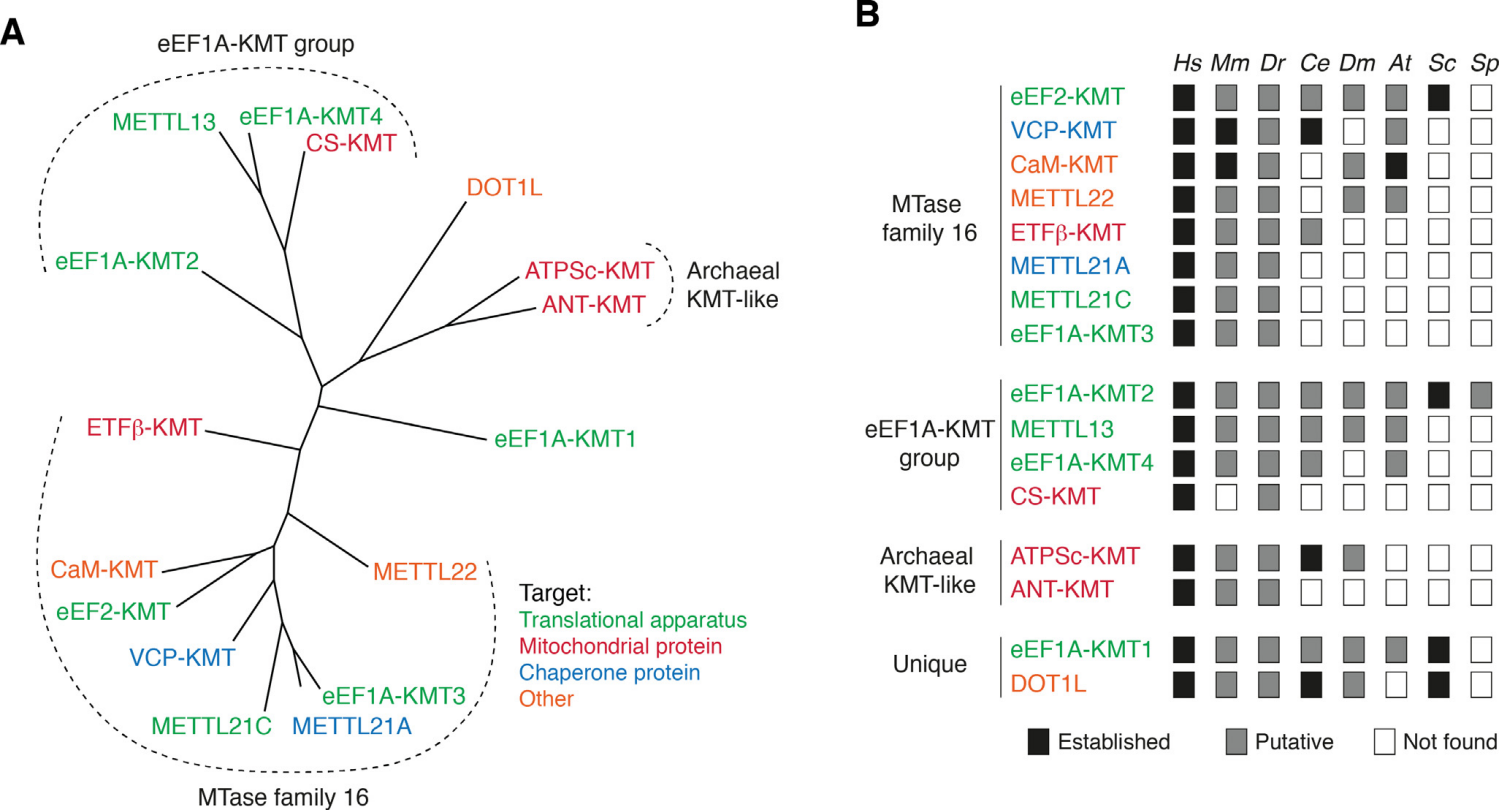
**Phylogenetic grouping and organismal distribution of human 7BS KMTs.***A*, phylogenetic tree of human 7BS KMTs. A sequence alignment of human 7BS KMTs was made using MAFFT (128) and then a tree generated and rendered using the PhyML and TREEDYN programs, respectively, found in the Phylogeny.fr package (129, 130, 131). *B*, organismal distribution of putative and established 7BS KMTs orthologues. For each human 7BS KMT, putative orthologues were identified by BLAST protein searches against the NCBI "Model organisms" database. A putative orthologue was defined as a protein that showed substantial sequence homology (expect value <10^-7^) to the relevant human KMT query, and, conversely, retrieved the query protein as the best human BLAST hit. Note: Such orthologue predictions are not entirely trivial: for example, by this criterion, the *S. cerevisiae* KMT Efm6 is a putative orthologue of human METTL21A, which targets Hsp70 proteins, but yeast Hsp70 proteins are not methylated at the relevant Lys residue, and Efm6 was found to methylate eEF1A (132). 7BS, seven-β-strand; At, *Arabidopsis thaliana*; Ce, *Caenorhabditis elegans*; Dm, *Drosophila melanogaster*; Dr, *Danio rerio*; eEF1A, eukaryotic elongation factor 1α; Hs, *Homo sapiens*; KMT, lysine-specific MTase; METTL, methyltransferase-like; Mm, *Mus musculus*; Sc, *Saccharomyces cerevisiae*; Sp, *Schizosaccharomyces pombe*.

There has been tremendous progress on identifying novel human 7BS KMTs during the last decade, and 16 such enzymes have now been characterized. We will keep the descriptions of the individual KMTs rather short, as several of them have already been covered by various review articles (23, 45, 46, 47). Interestingly, certain groups of proteins are overrepresented among the human 7BS KMT substrates, *i.e.*, components of the protein synthesizing machinery, mitochondrial proteins, and molecular chaperones. Therefore, the individual KMTs will here be described in the context of these groups; this is illustrated in Figure 2, which gives an overview of the human 7BS KMTs and their domain architecture. We have also generated a sequence-based phylogenetic tree to illustrate the degree of relatedness between the various human 7BS KMTs (Fig. 3*A*), showing that most of the KMTs belong to distinct groups of interrelated MTases, and that KMTs that methylate proteins from the same group or even the same protein (eEF1A) are often quite different. Figure 3*B* presents the organismal distribution of 7BS KMT orthologues in commonly used eukaryotic model organisms, and Table 1 gives an overview of the KMT targets, indicating the methylation site and state (me1, me2, and/or me3).

**Table 1:** Summary of human 7BS KMTs and their substrates

MTase^a^	Alisas(es)	Uniprot ID	Substrate	Lys^b^	State(s)	References
METTL21A	HSPA-KMT	Q8WXB1	HSPA1	561^c^	me3	(26, 30)
VCP-KMT	METTL21D	Q9H867	VCP	315	me3	(26, 33)
eEF1A-KMT1	N6AMT2	Q8WVE0	eEF1A^d^	79	me3	(28, 55)
eEF1A-KMT2	METTL10	Q5JPI9	eEF1A^d^	318	me3	(43, 56)
eEF1A-KMT3	METTL21B	Q96AZ1	eEF1A^d^	165	me1,2,3	(29, 37)
eEF1A-KMT4	ECE2	P0DPD7	eEF1A^d^	36	me3	(31)
METTL13	EEF1AKNMT, FEAT	Q8N6R0	eEF1A^d^	55	me2	(32, 34)
eEF2-KMT	FAM86A	Q96G04	eEF2	525	me3	(27, 61, 62)
METTL21C		Q5VZV1	AARS1	943	me1,2,3	(44)
ETFβ-KMT	METTL20	Q8IXQ9	ETFβ	200, 203	me1,2,3	(36, 41)
CS-KMT	METTL12	A8MUP2	CS	395	me3	(38, 42)
ATPSc-KMT	FAM173B	Q6P4H8	ATPSc	43	me3	(39)
ANT-KMT	FAM173A	Q9BQD7	ANT	52	me3	(40)
Dot1L	KMT4	Q8TEK3	H3	79	me1,2,3	(20, 21, 22)
CaM-KMT	CLNMT	Q7Z624	CaM	115	me3	(35)
METTL22		Q9BUU2	KIN	135	me3	(26, 94)

AARS1, alanyl-tRNA synthase 1; ANT, adenine nucleotide translocase; ATPSc, ATPS c-subunit; CaM, calmodulin; CS, citrate synthase; ECE2, endothelin-converting enzyme 2; eEF1A, eukaryotic translation elongation factor 1α; eEF2, eukaryotic translation elongation factor 2; ETF, electron transfer flavoprotein; HSPA, 70 kDa heat shock protein; KMT, lysine-specific MTase; METTL, methyltransferase-like; VCP, valosin-containing protein.

a These names are essentially identical to the current human gene names, except that the gene names are capitalized, italicized, without dashes, and not using greek letters, *e.g.*, *ETFBKMT*.

b Reflecting the prevailing practice in the scientific literature, the amino acid numbering refers to the canonical, nonprocessed UniProt-annotated protein, except for H3 and CaM, where the numbering refers to the protein without initiator methionine, and for ATPSc, where the numbering refers to the mature processed protein without the mitochondrial targeting sequence.

c Also targets the corresponding Lys residue in several other HSPA proteins, such as HSPA5, HSPA6, and HSPA8.

d Both eEF1A isoforms, eEF1A1 and eEF1A2, are methylated at the indicated sites.

Unfortunately, many KMT substrates reported in the literature are somewhat controversial and with limited experimental support. Thus, it has been pointed out that robust substrate assignment relies on the use of several independent methods (48). However, most of these issues concern various SET-domain KMTs, whereas less controversy has been associated with the 7BS KMTs (48, 49). Importantly, the substrates of the human 7BS KMTs have in most cases been independently identified in two or more studies, and the identifications are often corroborated by corresponding studies on yeast orthologues. In addition, the majority of the studies have used a combination of *in vitro* enzymology and KMT ablation in cells or tissues, followed by assessment of the substrate's modification status by protein mass spectrometry (MS), to firmly identify and validate substrates, thereby giving high credibility to the findings.

## The individual 7BS KMTs

### Chaperone-specific KMTs

Two human 7BS KMTs, namely the related valosin-containing protein (VCP)-KMT (METTL21D) and METTL21A, methylate molecular chaperones that use energy from ATP to facilitate the folding or unfolding of other proteins (26, 30, 33). They both belong to the so-called MTase family 16 (MTF16), which contains eight related human KMTs (Fig. 3*A*). Actually, an initial study also suggested that methylation of chaperones was a more general feature of the MTF16 members, as chaperone proteins were identified as interactants for several of these MTases (26). However, additional chaperone-targeting KMTs remain to be convincingly identified among the other MTF16 members.

#### VCP-KMT (METTL21D)

The essential and conserved molecular chaperone VCP (also known as p97) plays a crucial role in unfolding ubiquitinated proteins and is involved in a multitude of cellular processes, including protein degradation and quality control, as well as chromatin regulation and DNA repair (50). Two independent studies identified VCP as a binding partner of the previously uncharacterized MTase METTL21D, leading to the discovery that METTL21D catalyzes trimethylation of Lys-315 in VCP (26, 33). VCP contains two ATPase domains, D1 and D2, and forms a homohexameric ring, with Lys-315 located within the D1 domain, lining the inside of the ring. It was reported that METTL21D-mediated methylation diminished the ATPase activity of the D1 domain, but a direct effect of methylation on VCP chaperone activity remains to be shown (26). Interestingly, it was reported in 2011 that METTL21D promotes cancer metastasis but these potentially important findings have not been followed up by subsequent studies (51). Based on its substrate specificity, METTL21D has been renamed VCP-KMT (gene name: *VCPKMT*).

#### METTL21A (HSPA-KMT)

The Hsp70/HSPA proteins represent a ubiquitous family of ATP-dependent chaperones involved in a multitude of processes related to protein folding (52). Two independent studies found that the putative MTase METTL21A interacted with human Hsp70/HSPA proteins (26, 30) and it was demonstrated that METTL21A targets a lysine residue conserved between several of these, resulting in (mostly) trimethylation (26, 30, 49). Substrates of METTL21A include the heat- and stress-inducible Hsp70 protein HSPA1 (Hsp70), the constitutively expressed HSPA8 (Hsc70), and the endoplasmic reticulum-resident HSPA5 (GRP78/BiP) (26, 30). Interestingly, it was shown that METTL21A-mediated methylation of HSPA8 at Lys-561 diminished its ability to act as a chaperone for the Parkinson disease-associated protein α-synuclein (30).

### KMTs targeting the translational apparatus

A relatively large proportion of the human 7BS KMTs target the translational apparatus, and several of these KMTs are conserved in a wide range of eukaryotes, ranging from mammals to fungi and plants. With one exception (METTL21C), all of these methylate the two universally conserved translation factors eukaryotic translation elongation factor 1α (eEF1A; corresponding to bacterial EF-Tu) and eukaryotic translation elongation factor 2 (eEF2; corresponding to bacterial EF-G). eEF1A is a GTPase that brings aminoacylated tRNA to the translating ribosome, but a number of noncanonical functions have also been reported (53). Strikingly, five distinct 7BS MTases methylate specific lysine residues in eEF1A, somewhat analogous to how various SET-domain KMTs target the flexible tails of histones. In several cases, the gene KO of an elongation factor-specific KMT affected the overall protein synthesis or the translation of specific codons, indicating its functional importance (31, 32, 34, 37). It remains to be explored whether lysine methylations on eEF1A are tunable and subject to active regulation and whether they together may represent a "code" that controls protein synthesis. Note that two highly similar eEF1A paralogues, EF1A1 and eEF1A2, exist in mammals and that these are in the following collectively referred to as eEF1A.

#### eEF1A-KMT1 (N6AMT2)

Initially, this MTase was denoted N6AMT2, as it contains a so-called motif "Post II" (described in more detail in the subsequent section on “Grouping of human 7BS KMTs”) of sequence Asp-Pro-Pro-Tyr (DPPY), which is frequently found in MTases that mediate the *N*^6^-methylation of adenines in DNA or RNA (54). Somewhat surprisingly therefore, the putative *Saccharomyces cerevisiae* orthologue of N6AMT2 was demonstrated to be responsible for trimethylation of Lys-79 in eEF1A and denoted elongation factor methyltransferase 5 (Efm5) (55). Soon thereafter, human N6AMT2 was found to have the same biochemical activity as Efm5 and therefore renamed eEF1A-KMT1 (gene name: *EEF1AKMT1*) (28). So far, the functional significance of eEF1A-KMT1–mediated methylation of Lys-79 in eEF1A has not been investigated.

#### eEF1A-KMT2 (METTL10)

The MTase eEF1A-KMT2 (gene name: *EEF1AKMT2*), also known as METTL10, is conserved in many eukaryotes (23, 56). Its *S. cerevisiae* orthologue Efm4 was first shown to dimethylate Lys-316 in eEF1A (56, 57) and eEF1A-KMT2 was later demonstrated to trimethylate the corresponding lysine residue (Lys-318) in human eEF1A (43). The role of the eEF1A-KMT2–mediated methylation is so far unknown, but one study showed that the eEF1A-KMT2 function facilitates the replication of tombusviruses in plants and yeast (58).

#### eEF1A-KMT3 (METTL21B)

Two independent studies reported that the putative MTase METTL21B targeted eEF1A at Lys-165, introducing monomethylation, dimethylation, and trimethylation (29, 37). Since METTL21B represented the third enzyme-targeting eEF1A, it was redubbed eEF1A-KMT3 (gene name: *EEF1AKMT3*) (29, 37). Methylation of eEF1A at Lys-165 is highly variable, with Kme2/Kme3 being the most abundant forms in cancer cell lines and the nonmethylated form (Kme0) being predominant in normal tissues (37). Also, methylation at Lys-165 is dynamic and undergoes changes in response to various cellular stimuli, accompanied by corresponding changes in *eEF1AKMT3* mRNA levels (37). eEF1A-KMT3 is mainly found in the cytoplasm, but also in centrioles, where it colocalizes with eEF1A (37).

#### eEF1A-KMT4

This MTase was initially annotated as part of the endothelin-converting enzyme 2 (ECE2) due to overlapping sequences but was later found to represent a distinct protein (31). A robust MTase activity targeting K36 in eEF1A both *in vitro* and in cells was demonstrated, and the enzyme was renamed eEF1A-KMT4 (gene name: *EEF1AKMT4*) (31). The trimethylated form of K36 is the predominant form in different mammalian organs and, as other eEF1A lysine methylations, affects translation speed at distinct codons (31). A role in fine-tuning eEF1A function for optimal translation has been proposed for eEF1A-KMT4 (31). However, further studies are required to unravel the molecular mechanism behind eEF1A-KMT4–mediated modulation of eEF1A.

#### METTL13

Among the human 7BS MTases, METTL13 (also called eEF1A-KNMT or FEAT) is unique in harboring two MTase domains. The first (N-proximal) MTase domain is a KMT that dimethylates Lys-55 in eEF1A, whereas the second MTase domain trimethylates the N-terminus of eEF1A (32, 34). METTL13 is expressed ubiquitously and at relatively high levels (compared to other eEF1A-KMTs), especially in cancer cells where METTL13-mediated eEF1A Lys-55 methylation was shown to increase eEF1A GTPase activity and to enhance protein synthesis (34). Correspondingly, it was found that METTL13 modulates translation rates at specific codons *in vivo* and is required for efficient growth of Ras-driven cancers (32, 34). Additionally, elevated METTL13 and/or Lys-55 dimethylation levels have been found in various cancers and were in several cases linked to poor prognosis (59, 60). Therefore, METTL13 has been proposed as a therapeutic target and a clinical biomarker in cancer (34).

#### eEF2-KMT (FAM86A)

eEF2, which catalyzes the translocation of the nascent protein chain from the A-site to the P-site of the ribosome, is specifically methylated on Lys-525 by the MTase previously known as FAM86A and now renamed eEF2-KMT (gene name: *eEF2KMT*) (27). The corresponding lysine is methylated by the orthologous KMT Efm3 in yeast (27, 61, 62). This residue is located in close proximity to the “ribosomal accuracy center” (63), and indeed, the Efm3 KO strains displayed an increase in −1 frameshifting and sensitivity to several inhibitors of protein translation (27, 61). In addition, eEF2 appeared to be fully trimethylated in yeast and all analyzed mammalian cells and tissues, except the brain, where also lower methylation states were abundant (27). Taken together, this may suggest that eEF2 methylation has a constitutive function optimizing the accuracy of protein translation but may be subject to regulation in certain cell types of higher eukaryotes (27, 61, 62). Notably, in higher primates, eEF2-KMT is part of the FAM86 gene family that is contained within a tumor break-prone segmental duplication region, and 16 FAM86 sequences have spread to multiple genomic locations (64). Most of these are pseudogenes; however, two of them (besides eEF2-KMT), namely FAM86B1 and FAM86B2, are annotated as protein-coding (65), but their function remains to be investigated.

#### METTL21C

As indicated by its name, METTL21C forms, together with METTL21A, eEF1A-KMT3 (METTL21B), and VCP-KMT (METTL21D), a small subfamily of interrelated KMTs within MTF16 (Fig. 3*A*). Initially, two publications reported that METTL21C showed substrate specificities overlapping with its two chaperone-targeting relatives METTL21A and VCP-KMT (66, 67). One of these studies found that METTL21C methylated HSPA8 (*i.e.*, the substrate of METTL21A) (66), whereas the other reported that it targeted VCP (substrate of VCP-KMT) (67) but the experimental evidence was in both cases rather limited. Indeed, a subsequent, third study failed to observe *in vitro* activity of METTL21C on either HSPA8 or VCP (44). Instead, this study, supported by extensive biochemical and cellular evidence, found that METTL21C methylated Lys-943 in the alanyl-tRNA synthase 1 (AARS1), introducing a mixture of Kme1, Kme2, and Kme3 (44). Unlike other human 7BS KMTs, which are expressed rather ubiquitously, METTL21C is primarily expressed in skeletal muscle and is only found at low levels in most other tissues (44, 66, 67). Interestingly, two independent studies showed that METTL21C expression is restricted to slow muscles and that METTL21C KO resulted in decreased muscle performance (66, 67). In agreement with its role in modifying the translational apparatus, KO of METTL21C caused changes in the muscle proteome (67). It was noted that METTL21C-mediated methylation of AARS1, a component of the general protein synthesis machinery, does not align very well with METTL21C having a muscle-specific function (44). Thus, one should also consider the possibility that METTL21C may have other, muscle-specific substrates.

### Mitochondrial KMTs

Of the four 7BS KMTs that target mitochondrial proteins, two (ETFβ-KMT and CS-KMT) methylate soluble substrates in the mitochondrial matrix (36, 38, 41, 42). The other two (ATPSc-KMT and ANT-KMT) target proteins in the inner mitochondrial membrane and appear to be membrane-anchored (39, 40). Except for ATPSc-KMT and ANT-KMT, which show high sequence homology, the mitochondrial 7BS KMTs appear rather unrelated and have likely arisen independently in evolution (47).

#### ETFβ-KMT (METTL20)

The heterodimeric electron transfer flavoprotein (ETF) mediates electron transfer from various dehydrogenases that oxidize fatty acids, amino acids, and choline. Electrons are transferred to ETF:quinone oxidoreductase, which then mediates reduction of the mitochondrial ubiquinone pool (68). Two independent studies identified the putative MTase METTL20 as a mitochondrial KMT targeting the β-subunit of ETF, introducing monomethylation, demethylation, and trimethylation at two neighboring residues, Lys-200 and Lys-203 (36, 41). Based on its substrate specificity, METTL20 has been renamed ETFβ-KMT (gene name: *ETFBKMT*) (36). The targeted residues are located adjacent to ETF’s “recognition loop” involved in interaction with dehydrogenases (69) and their methylation was shown to inhibit ETF activity *in vitro* (36, 70). Accordingly, KO of *ETFBKMT* increased β-oxidation and heat production in mice, suggesting a role for ETFβ-KMT in regulating metabolism *in vivo* (71).

#### CS-KMT (METTL12)

CS catalyzes the synthesis of citrate from acetyl-CoA and oxaloacetate, which represents the rate-limiting step of the Krebs cycle. It was shown more than four decades ago, using protein sequencing, that mammalian CS is trimethylated at Lys-395 (13). Yet, only recently did two parallel studies identify the responsible mitochondrial KMT as METTL12, and the enzyme was redubbed CS-KMT (38, 42). Lys-395 in CS is constitutively trimethylated in mammalian tissues but is found hypomethylated in some cancer cell lines (38, 42). Methylation of CS inhibits its catalytic activity *in vitro*, and, interestingly, methylation by CS-KMT is prevented when CS adopts a “closed” conformation with bound oxaloacetate (38). It remains to be determined whether such metabolite-sensitive methylation of Lys-395 plays a functional role in regulating CS activity *in vivo*.

#### ATPSc-KMT (FAM173B)

Mitochondrial ATPS is a multiprotein complex that synthesizes ATP from ADP and phosphate, utilizing the proton-motive force generated during movement of electrons through the electron transport chain. The rotary element of ATPS contains the so-called c8-ring, which is assembled from eight ATPS c-subunits (ATPSc) and is embedded in the inner mitochondrial membrane (72). It was discovered nearly three decades ago that ATPSc contains a trimethyllysine at position 43 (12), but only recently was the mitochondrial KMT responsible for this modification identified as the hitherto uncharacterized MTase FAM173B, and consequently renamed ATPSc-KMT (39). Lys-43 is located on the matrix side of the “c8-ring” and was found invariably fully trimethylated in all *Metazoa* (73). Cells with ATPSc-KMT deficiency showed specific phenotypes, including defective ATPS assembly, reduced ATP synthesis, and reduced respiration (39). Interestingly, ATPSc-KMT overexpression has been implicated in promoting chronic pain in a mouse model (74).

#### ANT-KMT (FAM173A)

Adenine nucleotide translocase (ANT), also known as the ATP/ADP carrier, forms a protein channel across the inner mitochondrial membrane, transporting ADP into the matrix for ATP synthesis and ATP out of the matrix to fuel the cell (75). It was found more than four decades ago, by protein sequencing, that mammalian ANT is trimethylated at Lys-52 (76), but the mitochondrial KMT responsible for introducing this modification was identified only recently as FAM173A, and consequently renamed ANT-KMT (40). Lys-52 is a conserved residue present in all ANT isoforms, facing the matrix side, and was found predominantly trimethylated in ANT1, ANT2, and ANT3 from various mammalian cell lines and tissues (40, 77). Cells deficient in ANT-KMT showed increased mitochondrial respiration (40); however, the functional significance of ANT methylation at Lys-52 remains largely unknown.

### Other KMTs

Three of the human 7BS KMTs, namely DOT1L, CaM-KMT, and METTL22, methylate substrates outside the above-mentioned three groups that encompass most 7BS KMT substrates. DOT1L, which was the first mammalian 7BS KMT to be discovered, methylates histone H3. Somewhat surprisingly, however, given the abundance of lysine methylations on histones, none of the subsequently uncovered 7BS KMTs were found to target histones. It should be mentioned, though, that the 7BS MTase denoted HEMK2 or N6AMT1 was reported to methylate Lys-12 in histone H4 and therefore named KMT9 (78). However, this MTase was also reported to methylate the *N*^6^-position of adenines in DNA (hence the N6AMT1 name) (79). In addition, it represents a functional orthologue of the bacterial HemK protein (to which it also shows substantial sequence homology), catalyzing a universally conserved Gln methylation of a Gly-Gly-Gln (GGQ) motif in translational release factors (80). In a thorough biochemical study, the Gln MTase activity was shown to be considerably stronger than the KMT activity, whereas no DNA MTase activity was detected (81). Thus, further studies are required to properly assess the relevance of the KMT activity of HEMK2/KMT9/N6AMT1, which has not been included in our present 7BS KMT overview. The two other "unique substrate type" KMTs discussed in this section, CaM-KMT and METTL22, are both members of MTF16, which collectively targets a wide range of proteins.

#### Dot1L

The abundant lysine methylations in the flexible N-terminal tails of histones are introduced by SET domain KMTs. In contrast, the 7BS KMT DOT1L methylates Lys-79 in the globular part of histone H3 (20, 21, 22). DOT1L plays a key role in regulating chromatin structure and gene expression and is involved in several key cellular processes, such as telomeric silencing, DNA repair, and cell cycle regulation (82). Unlike the other human 7BS KMTs, which are "stand-alone" enzymes, DOT1L exerts its function in multiprotein complexes such as the so-called DotCom complex (83, 84). Many of the proteins in these complexes, including the DOT1L-binding protein AF9, are frequently found as fusion proteins with the mixed lineage leukemia 1 (MLL1) protein in leukemia (82). Correspondingly, DOT1L-mediated H3 Lys-79 methylation has been shown to be dysregulated in such leukemias (85). DOT1L has been extensively studied, and for further information on DOT1L you are referred to excellent review articles (82, 86).

#### CaM-KMT

The calcium (Ca^2+^) binding protein CaM binds to and regulates a number of enzymes in a Ca^2+^-dependent manner and is therefore an important mediator of cellular Ca^2+^ signaling. It was discovered more than four decades ago that mammalian CaM was trimethylated on Lys-115 (87, 88), and, in the following years, several studies investigated a CaM-KMT activity found in protein extracts from various mammalian tissues (87, 89, 90, 91). However, it was not until 2010 that the actual enzyme responsible for this activity was identified and denoted CaM-KMT (35). The functional importance of CaM methylation still remains rather elusive. However, one of the early studies reported that methylation diminishes the ability of CaM to interact with one of its targets, NAD kinase (92). Also, ablation or overexpression of the CaM-KMT orthologue in the plant *Arabidopsis thaliana* led to alterations in root length (93).

#### METTL22

Whereas all the other human 7BS KMTs target much-studied proteins with well-established functions in key cellular processes, METTL22 methylates KIN (aka KIN17), whose function is still poorly understood. METTL22 was shown to mediate trimethylation of Lys-135 in KIN *in vitro*, and the corresponding methylation was also found in mammalian cells (26, 94). Initially, KIN was implicated in several DNA-associated processes, such as DNA repair and replication (95), and in agreement with this, METTL22-mediated methylation of KIN was found to decrease the fraction of KIN associated with chromatin in the nucleus (94). However, more recent studies rather suggest a role for KIN in RNA processing, *i.e.*, in splicing and ribosome biogenesis (96). Thus, more insights on KIN function are needed to further understand the significance of its methylation.

## General discussion

### Grouping of human 7BS KMTs

Despite catalyzing the same reaction, *i.e.*, the methylation of the ε-amino group of a lysine residue (Fig. 1*A*), the 7BS KMTs represent a quite diverse set of enzymes within the 7BS superfamily. In terms of sequence similarity, they comprise three distinct groups of related MTases, as well as two "singlets," namely DOT1L and eEF1A-KMT1, that are not similar to any other human MTases (Fig. 3*A*), apart from the rather degenerate hallmark motifs involved in AdoMet binding. In particular, the members of each group share substantial sequence homology within a distinct motif, "Post II," which is localized at the end of the fourth β-strand (β4) and has been implicated in protein substrate recognition (23). The largest of the groups consists of eight members of MTF16, whereas the other major group consists of four KMTs, of which three target eEF1A, and is therefore denoted the "eEF1A-KMT group." Finally, a third group, denoted "Archaeal KMT-like," is formed by the two related mitochondrial KMTs ANT-KMT and ATPSc-KMT (aka FAM173A and FAM173B respectively), which show homology to KMTs from *Archaea* (39).

The low sequence similarity between several of the 7BS KMTs suggests that KMT activity has arisen independently several times throughout evolution and that the 7BS MTases have a propensity to readily evolve such activity. For example, the five KMTs that target eEF1A represent three distinct groups/types within the 7BS family, also illustrating that the same substrate protein can be methylated by unrelated KMTs.

### Organismal distribution and evolution

To identify 7BS KMT orthologues in common eukaryotic model organisms, we performed BLAST searches (97, 98). Putative and established orthologues are found in a wide range of eukaryotes (Fig. 3*B*). Some of the KMTs are found both in animals, plants, and fungi, *i.e.*, eEF1A-KMT1, eEF1A-KMT2, eEF2-KMT, and DOT1L, whereas others show a much more limited distribution. For example, CS-KMT is only found in a subset of vertebrates and is absent in many mammals (*e.g.*, rodents), and also eEF1A-KMT3 is limited to certain vertebrates. The number of 7BS KMTs typically increases with organismal complexity; whereas there are 16 such enzymes in humans, the budding yeast *S. cerevisiae* only has eight, with four of these having human orthologues (23). Taken together, this suggests that the 7BS KMT family has undergone extensive expansions, and, possibly, also losses, of paralogues throughout evolution, with some of the KMT functions acquired quite recently (Fig. 3, *A* and *B*).

Four of the human 7BS KMTs localize to mitochondria and three of these, namely ETFβ-KMT, ATPSc-KMT, and ANT-KMT, show strong sequence homology to KMTs from α-proteobacteria (39, 47, 70). According to the generally accepted endosymbiont theory, mitochondria once evolved from bacteria taken up by cells, and the bacterial lines that gave rise to mitochondria are most similar to today's α-proteobacteria (99). Thus, it is reasonable to assume that these three mitochondrial KMTs originate from bacteria.

### Substrate recognition and specificity

**Figure 4:**
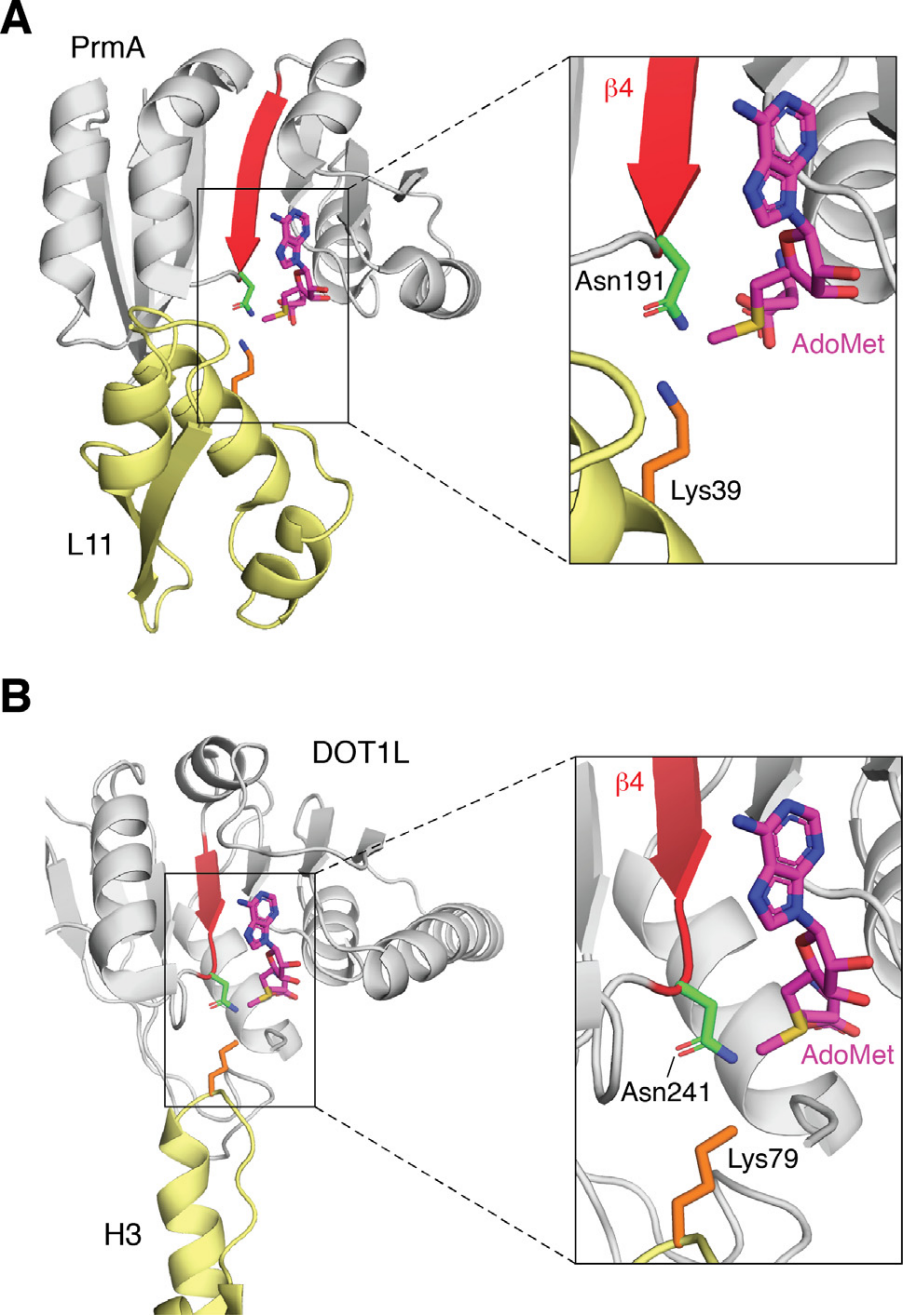
**The 7BS KMT active site.** Shown are the three-dimensional structures of the active site of the bacterial KMT PrmA (*A*) and mammalian DOT1L (*B*). The KMTs are shown in *gray*, except strand β4, which is shown in *red*, and with a key catalytic Asn residue (Asn191 in PrmA and Asn241 in DOT1L) in *green*. The substrate (L11 for PrmA and H3 for DOT1L) is shown in *yellow*, with the target lysine in *orange* (in the H3 structure, norvaline is a proxy for lysine). AdoMet is shown in *magenta*. The structural visualizations were made in PyMol version 2.4.1., using PDB entries 2NXN (PrmA–L11 complex), 2NXE (PrmA–AdoMet complex), and 6NJ9 (DOT1L–H3–AdoMet complex). 7BS, seven-β-strand; KMT, lysine-specific MTase.

For many years, bacterial PrmA was the only 7BS KMT for which structures of the complex between the enzyme and its protein substrate, in this case ribosomal protein L11, were available (100). However, a cryo-EM structure of DOT1L in complex with its substrate, Lys-79 in histone H3 in the context of a nucleosome, was published in 2019 (101). This allows for a comparison between the two KMT-substrate complexes. In the PrmA-L11 structure, Asn-191 in PrmA interacts with Lys-39 in L11, the target of methylation. Interestingly, a comparison of the structures of DOT1L, PrmA, and human VCP-KMT showed that Asn-191 in PrmA, which is part of motif "Post II" and localized to the end of strand β4, is near-superimposed with corresponding Asp/Asn residues in DOT1L and VCP-KMT and with a similar orientation relative to the cosubstrate AdoMet (23). A comparison of the KMT-substrate structures for DOT1L and PrmA indeed demonstrates that the targeted lysine shows a very similar orientation relative to AdoMet and to the key Asn residue (Fig. 4). This may suggest that the targeted lysine residue is similarly positioned, relative to AdoMet and the 7BS-fold, also in other 7BS KMTs.

KMTs of the SET family typically recognize, in their respective substrates, a linear sequence encompassing the target lysine and its surrounding residues, and several of these KMTs target multiple substrates containing a specific consensus sequence. In contrast, the 7BS KMTs appear to recognize primarily the three-dimensional features of a folded substrate and to a lesser extent the local sequence around the methylation site. For example, replacing the residues surrounding the targeted lysine in VCP with alanines did not affect VCP-KMT–mediated methylation (33). Moreover, various chimeric VCP proteins, where the target lysine and surrounding residues had been replaced by lysine-containing histone-derived sequences (of identical length and lysine positioning), were subject to VCP-KMT–mediated methylation, both *in vitro* and in cells (102). Many of the 7BS KMT substrates were initially identified as 7BS KMT interactants, thus showing, in their folded state, a high affinity for the respective MTases, and all the human 7BS KMTs have, so far, been found to target only a single substrate (or a group of highly similar substrates). This indicates that the 7BS KMTs recognize the three-dimensional structure of their substrates in a highly specific manner. However, it should be mentioned that the methods used for uncovering KMT substrates are biased toward identifying abundant proteins and that most of the 7BS KMT substrates found thus far are indeed highly abundant. Therefore, it should not be excluded that additional, less abundant, substrates exist.

### A distributive mode of MTase action

A KMT can introduce up to three methyl groups on a lysine residue (Fig. 1*A*), with the maximal/final methylation state determined by the geometry of the KMT active site. Some KMTs, especially members of the SET-domain family, are processive, meaning that they are capable of introducing more than one methyl group per binding event. This is because exchange of the AdoHcy for AdoMet can occur without the protein substrate dissociating from the KMT. Typically, processive KMTs will therefore generate one specific Lys methylation state, *e.g.*, dimethylation or trimethylation. In contrast, the so-called distributive KMTs must dissociate from the substrate after one round of methylation and may in the next round bind to and methylate another substrate molecule, typically resulting in a stochastic mixture of methylation states. Overall, it appears that most, if not all, 7BS KMTs are distributive enzymes, because incubation of substrate with limiting enzyme concentrations has invariably (when this was assessed) given a mixture of methylation states, and such mixed methylation states have also been observed in cells and tissues (33, 38). However, for some 7BS KMTs, such as ATPSc-KMT and ANT-KMT, the corresponding substrates were exclusively found in their fully trimethylated states in cells and tissues (39, 40). However, this does not necessarily mean that these enzymes are processive but may merely reflect that there is a high level of KMT activity, thus pushing the reaction all the way toward trimethylation.

### Effects of cofactors/ligands and substrate configuration on KMT activity

Many 7BS MTases require accessory protein factors to manifest their enzymatic activities; this is particularly common among MTases that target RNA (103). In contrast, the majority of human 7BS KMTs appear to be stand-alone enzymes capable of catalyzing their respective reactions without the involvement of accessory proteins. Accordingly, for most of the KMTs, very robust *in vitro* activity was observed when recombinant enzyme, protein substrate and AdoMet were incubated together. Also, the majority of human 7BS KMTs are rather small proteins, devoid of domains putatively involved in protein–protein interactions; 13 out of the 16 human 7BS KMTs are between 218 and 330 amino acids long (Fig. 2). One exception in this respect is DOT1L, which is part of the so-called DotCom multiprotein complex and contains several different domains involved in its recruitment to chromatin (83, 84). Moreover, the intrinsic activity of the DOT1L catalytic domain is low, and it has been shown that efficient DOT1L-mediated methylation requires interaction with ubiquitinated histone H4 (104, 105). This causes conformational changes that place DOT1L and the target lysine (K79) in an orientation favorable for methylation (101).

Most 7BS KMTs show robust *in vitro* activity on recombinant *Escherichia coli*‒expressed substrates, but in some cases MTase activity was only observed with a specific configuration of the substrate. VCP forms a ring-shaped hexameric structure with the target lysine located inside the ring, and efficient VCP-KMT–mediated methylation was only observed with a VCP deletion mutant deficient in oligomer formation (33). Also, eEF1A-KMT3 was unable to efficiently methylate recombinant eEF1A but was very active on eEF1A in a cell extract, indicating that efficient methylation of eEF1A requires the full eEF1 complex or, alternatively, specific posttranslational modifications (37). Moreover, enzymatic activity of DOT1L is only observed when its target histone H3 is part of a nucleosome (20). Several of the human 7BS KMT substrates bind small ligands or cofactors, and there are several examples that methylation is affected by such binding. Methylation of the GTPase eEF1A on Lys-165 and Lys-36, which is mediated by eEF1A-KMT3 and eEF1A-KMT4, respectively, requires the presence of GTP, whereas methylation on Lys-55 by METTL13 was slightly diminished by GTP (31, 32, 37). Furthermore, methylation of CS by CS-KMT was blocked by the presence of the CS substrate oxaloacetate, which induces a closed conformation in the CS structure (38). Conceivably, modulation of KMT-mediated methylation by small ligands may play an active role in regulating the substrate's activity, but this needs to be investigated further.

### Dynamics and regulation

Lysine methylations on the flexible tails of histone proteins play important regulatory roles, and they are also highly dynamic, as they can be actively reversed by a set of highly specific demethylases. In contrast, none of the methylations mediated by the 7BS KMTs appear to be actively demethylated. Also, many of these methylations are often of high occupancy, typically full trimethylation, and showing little variation between different cells and tissues. This may suggest that such methylations optimize the performance of the substrate protein in a constitutive manner, rather than dynamically regulating it.

However, there are some examples that 7BS KMT‒catalyzed methylations can be dynamic and of regulatory importance. For example, DOT1L regulates the cell cycle, and the resulting methylation of Lys-79 in histone H3 showed variations throughout the different phases of the cell cycle (106). Also, the eEF1A-specific KMT eEF1A-KMT3 (METTL21B) is subject to regulation, and a considerable variability in the corresponding methylation of Lys-165 in eEF1A was observed (37). When cells were exposed to various stressors such as serum starvation, contact inhibition, and ER stress, eEF1A-KMT3 was transcriptionally upregulated, with a concomitant increase in eEF1A-K165 methylation (37). Moreover, EFTβ-KMT–mediated methylation of the mitochondrial electron-transfer protein ETFβ displayed a wide range of methylation states *in vitro* and *in vivo*, suggesting a dynamic regulatory role also for this methylation event (36).

Finally, one should not exclude the possibility that other 7BS KMTs may play important roles in dynamic regulation of protein function, although there is so far little experimental support for this. Virtually all the relevant experiments yet performed have used unstressed cells growing in rich cell culture media or well-fed animals living under controlled laboratory conditions. Thus, these KMTs may conceivably regulate protein function under the more variable and stressful conditions faced by wild organisms living under evolutionary pressure.

### Molecular-level functional effects

For several of the human 7BS KMTs, methylation showed inhibitory effects on the substrate protein and/or relevant processes. CS-KMT–mediated methylation diminished CS activity, and METTL21A-mediated methylation of the human Hsp70 protein HSPA8 reduced its ability to prevent α-synuclein fibril formation (30, 38). ETFβ-KMT–mediated methylation of ETFβ decreased its ability to extract electrons from various mitochondrial dehydrogenases, and corresponding metabolic effects were observed in ETFβ-KMT KO mice (36, 71). Moreover, ANT-KMT KO cells showed increased mitochondrial respiration, suggesting that unmethylated ANT is more active than its methylated counterpart (40). In contrast, there are also examples that 7BS KMT–mediated methylation increases the substrate's activity. METTL13-mediated methylation of eEF1A increased its GTPase activity, as well as overall protein synthesis (34). Also, results with ATPSc-KMT KO cells indicate that methylation of ATPSc promotes the assembly and activity of the ATPS complex (39). In summary, 7BS KMT–mediated methylation appears to inhibit the substrate protein's function in some cases and enhance it in others.

Some more general functions for protein lysine methylation have also been proposed and these are clearly also relevant regarding the 7BS KMTs. The methylation of a lysine residue will prevent its ubiquitination, and it has therefore been speculated that methylation is a way of increasing protein stability by hindering lysine ubiquitination and subsequent proteasomal degradation (107). However, this has yet to be demonstrated for any specific 7BS KMT substrate, *i.e.*, there are to our knowledge no examples that 7BS KMT KO decreases the stability of the corresponding substrate protein. However, the target site for METTL21A methylation in HSPA1, K561, has also been shown to be a target of ubiquitination, suggesting that METTL21A-mediated methylation may promote HSPA1 stability (108).

Free (nonproteinaceous) trimethyllysine (TML) is the precursor for the cellular synthesis of carnitine, which plays an important role in fatty acid transport into mitochondria. TML cannot be synthesized directly, and it is believed that degradation of Kme3-containing proteins is the primary source of free TML in mammals (109). However, it has also been suggested that TML may be provided through vegetables in the diet (110). Given the high abundance of many of their substrates, it is likely that the 7BS KMTs, collectively, play an important role in providing TML for carnitine synthesis. However, it appears less likely that this is the primary “raison d'être” for any individual 7BS KMT.

### Organismal effects and disease relevance

Many of the human 7BS KMTs show a scattered distribution within the eukaryotic kingdom (Fig. 3*B*), whereas the targeted lysine residue is generally much more conserved. Thus, the relevant substrate protein will exist in an unmethylated state in many organisms, suggesting that methylation is not strictly required for the basic protein function but rather serves a regulatory or modulating purpose. Correspondingly, the functional effects caused by 7BS KMT–mediated methylations appear in many cases rather modest and subtle, both at the level of the substrate proteins, cellular/physiological processes, and the full organism. For example, KO of METTL21A and VCP-KMT in the mouse did not give any overt phenotype, and genetic data from the Islandic population indicate that natural human KO individuals of ETFβ-KMT are healthy and unaffected (111, 112, 113). This agrees well with these KMTs being limited to multicellular eukaryotes and showing a somewhat scattered distribution within the animal kingdom. In the case of CaM-KMT, which is also only found in multicellular eukaryotes, the corresponding KO mouse was viable but did show functional defects related to the brain, muscles, and mitochondria (114). For DOT1L, which shows a more widespread evolutionary distribution and is present also in unicellular eukaryotes such as the budding yeast *S. cerevisiae*, the KO mouse was not viable (115). Thus, it may be speculated that 7BS KMTs with a broader evolutionary distribution serve a more critical function. It will therefore be of interest to investigate the phenotypic effects of mouse KO of eEF2-KMT, eEF1A-KMT1, and eEF1A-KMT2, all of which are present in both unicellular and multicellular eukaryotes.

Seven of the human 7BS KMTs target components of the translational machinery, such as the elongation factors eEF1A and eEF2. For several of these KMTs, it was demonstrated that gene KO in human cells affected the rate and/or fidelity of mRNA translation. mRNA translation is a highly optimized process, and it has been demonstrated that altering translation rates through altering modifications on the translational machinery can lead to aggregation of proteins (116). Also, chaperones such as VCP and Hsp70 proteins are key to ensure appropriate protein folding and turnover, thereby preventing protein aggregation. Since the formation of protein aggregates is a hallmark of many types of neurological diseases, one may therefore speculate that defects in 7BS-KMT–mediated methylation of chaperones and translation factors may lead to such diseases.

For some of the human 7BS KMTs a more direct association with disease has been reported. DOT1L plays an important role in the epigenetic dysregulation occurring in mixed lineage leukemia (117, 118). Also, several other 7BS KMTs have been implicated in cancer, *i.e.*, VCP-KMT (51), METTL13 (34, 59, 119), and eEF1A-KMT3 (METTL21B) (120). Interestingly, genome-wide studies have linked ATPSc-KMT to chronic pain, and ATPSc-overexpression was shown to promote chronic pain in a mouse model (74, 121). METTL21C has been linked to musculoskeletal diseases, *i.e.*, to bone mineral density disorder, osteoporosis, and sarcopenia (122, 123).

### Notes on the 7BS MTase, METTL, and KMT nomenclature

In 2010, Petrossian *et al*. published an overview of the human "methyltransferasome", *i.e.*, a catalogue of established and putative human MTases, where the latter were identified bioinformatically through sequence searches and secondary structure predictions (19). More than 200 human MTases were reported and the 7BS MTases constituted the large majority of these, *i.e.*, 131 proteins (19). At the time, many of the MTases only existed as theoretical proteins, *i.e.*, ORFs predicted from the human genome sequence, and had been named accordingly. Following this work, many of the identified 7BS MTase ORFs that encoded uncharacterized proteins were renamed as methyltransferase-like proteins (METTLs). For example, C12orf72 and C16orf68 were renamed METTL20 and METTL22, respectively. However, many human 7BS MTases were not given a METTL name, *i.e.*, those that had already been functionally characterized, such as DOT1L, the DNA methyltransferases, and the tRNA MTase ALKBH8, as well as several putative MTases that had already been given a "non-ORF" name, such as FAM86A (now eEF2-KMT), FAM173A (ANT-KMT), and FAM173B (ATPSc-KMT). Therefore, it is important to note that the METTLs do not encompass a clearly defined MTase group, but that the ∼34 7BS MTases that were given a METTL name, constitute a rather arbitrary and historically selected subgroup within the 7BS MTases. Still, the METTLs are often referred to as a distinct MTase group in the literature, and specific review articles and omics studies have even been devoted to this subset of the 7BS MTases (124, 125, 126, 127).

For the 7BS KMTs, a new and more descriptive nomenclature was initiated in 2010, when the long-sought KMT-targeting calmodulin was identified and named CaM-KMT (gene: *CAMKMT*) (35). Since then, most of the novel 7BS KMTs have been given a similar, "substrate-based" name, which, due to the above-mentioned shortcomings of the METTL nomenclature, appears to be a good choice. Thus, most of these renamings have now been approved by the Human Genome Nomenclature Committee. However, some 7BS KMTs have retained their METTL names, *i.e.*, METTL13 (which also has non-KMT MTase function) and METTL22, as well as METTL21A and METTL21C (both names have already been used quite extensively in the literature).

## Conclusion and future perspectives

In recent years, there has been tremendous progress on identifying novel human 7BS KMTs. A little more than a decade ago, only one such enzyme (DOT1L) was known, but 16 7BS KMTs have now been discovered and characterized. In parallel, the number of uncharacterized human 7BS MTases has decreased dramatically and now this group does not encompass any obvious putative KMTs. However, for many of the reported lysine methylation events, the responsible KMTs still remain elusive, and one should not exclude the possible existence of additional 7BS KMTs that only show limited similarity to the ones already identified. Still, the major future challenges will be to understand the biological and physiological functions of the known 7BS KMTs.

## Conflict of interest

The authors declare that they have no conflicts of interest with the contents of this article.
